# Age-related hearing loss in older adults: etiology and rehabilitation strategies

**DOI:** 10.3389/fnins.2024.1428564

**Published:** 2024-10-01

**Authors:** Qinzhi Zheng, Zhuo Xu, Nan Li, Yueying Wang, Ting Zhang, Jiapeng Jing

**Affiliations:** ^1^Department of Rehabilitation, China-Japan Union Hospital of Jilin University, Changchun, China; ^2^Rehabilitation Therapeutics, School of Nursing, Jilin University, Changchun, China

**Keywords:** age-related hearing loss, rehabilitation, elderly, presbycusis, review

## Abstract

Age-related hearing loss (ARHL) is a prevalent sensory organ disorder among elderly individuals that significantly impacts their cognitive function, psychological well-being, and ability to perform activities of daily living. As the population ages, the number of ARHL patients is increasing. However, the Audiological rehabilitation (AR) status of patients is not promising. In recent years, there has been an increasing focus on the health and rehabilitation of elderly individuals, and significant progress has been made in researching various age-related disorders. However, a unified definition of ARHL in terms of etiology and rehabilitation treatment is still lacking. This study aims to provide a reference for future research on ARHL and the development of AR strategies by reviewing the classification, etiology, and rehabilitation of ARHL.

## 1 Introduction

Age-related hearing loss (ARHL), also known as presbycusis, is a condition that is characterized primarily by a decline in hearing ability that worsens with age. The condition typically begins with a loss of high-frequency hearing and progresses to include medium-and low-frequency hearing loss. ARHL is one of the most common age-related conditions affecting older adults, second only to cardiovascular and arthritic diseases. It is also one of the most common sensory disorders. Research indicates that ARHL is linked to the onset of serious mental health conditions, falls, cognitive impairment and Alzheimer’s disease (Livingston et al., 2017; Wilson et al., 2017; Lin et al., 2011; Livingston et al., 2020; Bowl and Dawson, 2019). In addition, approximately half of the people over 70 years of age suffer from social isolation due to the severe impact of ARHL on daily communication, further exacerbating anxiety, depression, and other psychological disorders among people with ARHL (Bowl and Dawson, 2019).

On the basis of the *Global Burden of Disease2019 (GDB 2019)* data, a significant number of individuals require rehabilitation due to hearing loss. Approximately two-thirds of these individuals are aged 60 years or older, and half of the total number of patients suffer from severe or greater hearing loss (Cieza et al., 2021). However, although hearing aids (HAs) and hearing assistive devices have been shown to be effective in improving the quality of life of ARHL patients, surveys have shown that the HAs ownership rate in the HAs-fit population is only approximately 25% and that approximately 30% of these patients still own but do not use an HA (Walling and Dickson, 2012). Furthermore, the dearth of resources allocated to AR, coupled with the uneven distribution of these resources, renders it challenging for the majority of the population with hearing impairments to access quality rehabilitation services. The purpose of this review is to illuminate the etiology and rehabilitation strategy of ARHL, with the goal of advancing our understanding, prevention, and rehabilitation of ARHL.

## 2 Methods

PubMed and Web of Science were searched from database inception to August 31, 2024, using the MeSH subject terms *Hearing Loss* and *Presbycusis*, and the general subject term *Age Related Hearing Loss*, with filters for *English-speaking*, *older adults* (60 years and older), and *humans*. Animal experiments, cellular experiments, reviews and meta-analyses related to ARHL were provided by each author and are discussed uniformly. The authors also consulted the known literature and policy statements.

## 3 Classification and risk factors for ARHL

### 3.1 Classification

**Figure 1:**
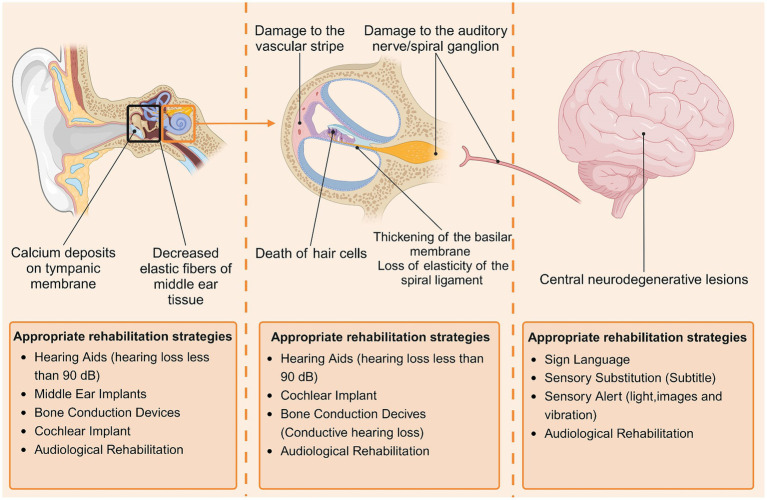
Major histopathologic changes in patients with ARHL and corresponding rehabilitation strategies. Histopathologic changes in patients with ARHL are found mainly in the middle ear (tympanic membrane, tympanic chamber), inner ear (cochlea, auditory nerve), and central nervous system, and different types of pathologic changes lend themselves to different rehabilitation strategies. By Biorender.

The currently recognized clinical subtypes of ARHL are divided into the following six categories: (1) sensorineural ARHL, characterized by the death of hair cells outside the cochlea; (2) neurological ARHL, characterized by damage to the auditory nerve/spiral ganglion; (3) vascular ARHL, characterized by damage to the vascular stripe of the lateral wall of the cochlea; (4) conductive/mechanical ARHL, characterized by thickening of the basilar membrane and loss of elasticity of the spiral ligament; (5) central ARHL, characterized by central neurodegenerative lesions; and (6) mixed ARHL with two or more lesions occurring simultaneously. The histopathological changes involved in different types of ARHL vary, and patients should choose the appropriate rehabilitation strategy for their lesions (Figure 1).

Owing to the complexity of the etiology and pathomechanisms of ARHL, clinically all types of ARHL are usually present at the same time, and most patients present with mixed ARHL (Bowl and Dawson, 2019).

### 3.2 Risk factors

#### 3.2.1 Genetic factors

Past genetic studies have shown that congenital and early-onset hearing loss has a genetic component, which appears to be a characteristic of ARHL (Azaiez et al., 2018). Early investigations suggested that approximately 50% of hearing loss in older relatives may be genetically related (Keithley, 2020). In recent years, it has become increasingly recognized that ARHL is likely associated with a polygenic genetic predisposition, and many scholars have investigated this susceptibility to the disease (Wang and Puel, 2020). Studies have shown that the pathogenesis of ARHL is genetically related in 25–75% of cases (Viljanen et al., 2007; Christensen et al., 2001; Gates et al., 1999). A genome-wide association study (GWAS) is a research method that searches for genetic factors associated with complex diseases via genome-wide high-density genetic marker typing of large-scale population DNA samples; this method was first successfully used by Klein et al. (2005). In 2005 in their study of age-related macular degeneration, and in recent years, researchers have screened many candidate genes for ARHL via this method. A GWAS of middle-aged and older adults aged 40–69 years reported that the heritability of age-related hearing loss (ARHL) was estimated to be between 35 and 55% (Wells et al., 2019). Glutamate metabotropic receptor 7 (GRM7) is a G protein-coupled receptor whose activation is associated with neurotransmitter inhibition, and the GRM7 gene is a candidate gene that is currently receiving increased attention. Studies have shown that genetic variation in GRM7 is likely associated with the development of ARHL and invisible hearing loss and that the overexpression of GRM7 may lead to the inhibition of neurotransmission between hair cells and synapses. In addition, the GRM7 SNP rs11928865 (TT) gene is more common in specific types of Chinese elderly Han Chinese male patients with ARHL, and this finding may aid in the clinical screening and classification of ARHL patients (Friedman et al., 2009; Newman et al., 2012). In addition, the deletion of the mitochondria-associated gene mtDNA 4,977-bp is believed to be a contributing factor in the development of ARHL (Han et al., 2019). This deletion impairs the function of the mitochondrial oxidative phosphorylation process, leading to the formation of bioenergetically deficient cells (Uchida et al., 2014). In addition, studies have identified genes such as ahl1, ahl2, ahl3, and granular head-like 2 (GRHL2) in ARHL mice; miR-34a in the mouse auditory cortex; the silent information regulator 3 (sirtuin3) gene; and the p.V37I mutation in the gap junction protein-related gene (GJB2), as well as the clinical manifestations of the disease, which are very similar to those of ARHL. The genes associated with DFNA5, a noncomprehensive sensorineural deafness that is very similar to ARHL, are all likely genetic factors for ARHL (Wang and Puel, 2020; Chen et al., 2022; Huang et al., 2017). Notably, although certain genes have been shown to be significantly correlated with ARHL, ARHL still occurs as a result of multiple genes acting together to reach a certain threshold rather than a single gene.

#### 3.2.2 Deterioration with age

Aging is the gradual accumulation of harmful biological changes that cause a progressive decline or loss of tissue and organ function over time (Wang and Puel, 2020). The human body’s ability to circulate energy, its metabolism, and its antioxidant capacity weakens as it ages, resulting in impaired energy circulation, increased free radicals, and accelerated apoptosis in the cochlea, which diminishes hearing. Aging causes changes in the cochlear tissue structure, including thickening of the basilar membrane, weakening of the elasticity of the spiral ligaments, and narrowing and hardening of the blood vessels, leading to impaired sound conduction, ischemia, and hypoxia in the cochlea. Hearing thresholds decline at an accelerated rate with age, even in the absence of noise exposure or other hearing-affecting diseases (Bielefeld et al., 2010).

#### 3.2.3 Noise exposure

Noise exposure is widely acknowledged as a major contributing factor to ARHL in elderly individuals. Research studies dating back to the 1960s and 1970s have consistently provided evidence supporting this view. Research has shown that the Mabaan tribe, which has lived in the Sudanese desert for generations, has hearing conditions similar to those of people in the same age group worldwide. However, the tribe members maintain relatively healthy hearing in old age, which is closely linked to the low-noise environment in which they live (Rosen et al., 1962). Recent clinical studies have shown that long-term exposure to noise accelerates the development of ARHL (GBD 2019 Hearing Loss Collaborators, 2021). Animal experiments also support this view. Kujawa and Liberman (2019) reported that noise exposure can damage the stereocilia bundles of mice, guinea pigs, cats, and other animals. Zhao et al. (2020) demonstrated that noise exposure is one of the significant factors leading to hearing loss and cochlear pathology in mice. Notably, mice can experience hearing loss beyond normal levels in old age, even when exposed to acoustic environments that cause only transient hearing threshold shifts (Keithley, 2020). In recent years, new studies have proposed various therapeutic targets or approaches for the treatment of noise-induced hearing loss, e.g., epidermal growth factor inhibitors have shown promising efficacy in mouse and zebrafish models, superparamagnetic iron oxide nanoparticle assembly (SPIOCA) can remodel gut dysbiosis to treat noisy hearing loss, and nicotinamide encapsulated by personalized porous gelatin methacrylamide can effectively enhance drug delivery efficiency to treat noisy hearing loss. Unfortunately, however, there are no FDA-approved drugs for the treatment of noise-related hearing loss, meaning that there are no specific therapeutic targets or effective drug delivery strategies. Therefore, more research is needed in the future (Vijayakumar et al., 2024; Guo et al., 2024; Feng et al., 2024).

#### 3.2.4 Metabolic disease

Metabolic diseases are closely related to the development of ARHL. Ge et al. (2024) confirmed that hypertension, diabetes, hyperlipidemia, and hyperuricemia are risk factors for ARHL using a new screening tool. Moreover, research has shown that there is a close relationship between hyperglycemia and hyperlipidemia and the progression of hearing loss, as demonstrated by animal experiments and meta-analyses (Li et al., 2023; Nguyen et al., 2022). Among these factors, hyperglycemia is the most commonly cited and studied risk factor for hearing loss. A large cross-sectional study of more than 37,000 individuals revealed that the prevalence of hearing loss increases with age and the presence of diabetes and that the prevalence of hearing loss in people with diabetes is more than twice as high as that in people without diabetes (Oh et al., 2014). It has been demonstrated that hyperglycemia induces damage to mitochondrial DNA, further impairing oxidative phosphorylation and ATP production, and that this damage accelerates the aging of high-energy-demanding tissues such as the cochlea and kidneys (Forbes and Thorburn, 2018). Studies in rats with advanced type 2 diabetes revealed histopathological changes consistent with the vascular changes observed in the human inner ear and correlated with the course of diabetes (Ishikawa et al., 1995). Notably, current research on older patients with hearing loss still lacks strong evidence to establish a direct link to diabetes and does not exclude the interference of other risk factors, such as noise exposure.

#### 3.2.5 Ototoxic drugs

Certain medications, including cisplatin drugs, aminoglycosides, NSAIDs, and loop diuretics, are known to be toxic to the cochlea (Nieman and Oh, 2020). According to Joo et al. (2020) study, individuals taking labeled diuretics had a 33% greater risk of hearing loss over a 40-year period, whereas those taking NSAIDs had a 45% greater risk of progressive hearing loss than those who did not take them. Li et al. (2020) and Li et al. (2016) study confirmed that continuous injection of gentamicin in CBA/J mice caused a reduction in cochlear ribbon synapses, impaired hearing and downregulated the expression of fibroblast growth factor 22 (FGF22). In contrast, the administration of exogenous FGF22 attenuated the ototoxicity of gentamicin while protecting the hearing of the mice. A new study identified a potential therapeutic molecule, piplartine, that protects hearing function in mice without interfering with the antimicrobial effects of aminoglycosides. The rationale is that piplartine prevents kanamycin from entering mouse ear tissue by increasing the expression of TRPV1, thereby preventing kanamycin-induced hair cell loss and protecting the hearing of mice (Zallocchi et al., 2024). Cisplatin is a chemotherapeutic agent commonly used to treat solid tumors, including ovarian cancer. However, it is also known for its cumulative toxic effects in humans. According to Steyger et al. (2018) study, cisplatin-induced DNA damage and activation of apoptotic processes may be the cause of cisplatin-induced hearing loss, and cisplatin inhibitors have been shown to be effective in the treatment of hearing loss. Due to the weakened metabolic capacity of the elderly body, it takes longer to metabolize ototoxic drugs. This may be one of the reasons for the increased incidence of hearing loss in elderly individuals taking ototoxic drugs.

#### 3.2.6 Other factors

In addition to the aforementioned factors, several other variables have been linked to ARHL development. Qian et al. (2023) reported that alcohol consumption is one of the risk factors for hearing loss and that limiting alcohol consumption can help prevent hearing loss. Furthermore, hearing loss was found to be associated with factors such as educational experience, location, economic level, and sex (Li et al., 2023).

## 4 Pathematology and pathogenesis

### 4.1 Pathematology of ARHL

Studies on the morphology of the human temporal bone during life and after death have shown that ARHL is caused primarily by damage to cochlear sensory cilia, atrophy of the cochlear vascular stripe, thickening of the basilar membrane, damage to the spiral ligament, and degenerative lesions of the auditory nerve/spiral ganglion. This is supported by numerous animal studies (Bowl and Dawson, 2019; Schuknecht and Gacek, 1993; Schuknecht, 1955). Notably, animal-based studies have also shown some experimental phenomena. Gates and Mills (2005) reported that the most obvious manifestation of ARHL development in animals was vascular pattern atrophy. In addition, auditory nerve degeneration is the first pathological change, and synaptosis and glutamate between hair cells and the auditory nerve and decreased glutamate secretion may be important reasons for early hearing loss in ARHL (Liberman and Kujawa, 2017; Sun et al., 2021; Kujawa and Liberman, 2006; Qian et al., 2021).

### 4.2 Mitochondrial activity and energy metabolism

**Figure 2:**
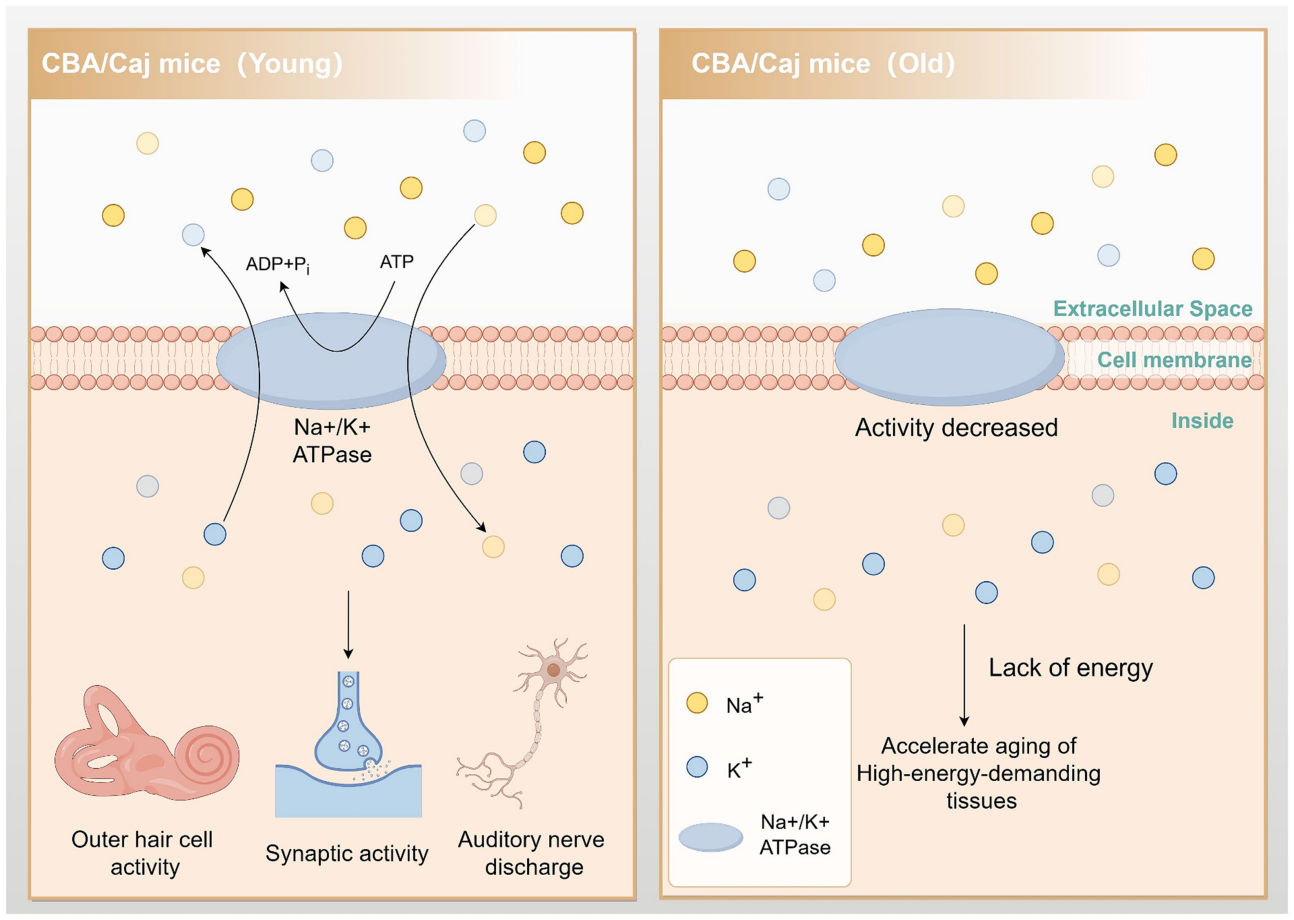
Reduced Na/K-ATPase activity leads to accelerated cochlear aging. In aging CBA/Caj mice, Na/K-ATPase activity is reduced by approximately 80%. This change impedes cochlear hair cell movement, synaptic activity, and auditory neuron firing, accelerating the aging of these energy-demanding tissues. By Figdraw.

Mitochondria are intracellular organelles responsible for energy production and are the primary source of endogenous reactive oxygen species (ROS). They play a vital role in the activities of all tissue cells, including the cochlea. The inner ear uses energy to maintain the cochlear potential generated by the vascular striatum. This energy is necessary to assist in the movement of outer hair cells, carry out synaptic activity, and maintain spontaneous and sound-driven discharges of auditory neurons in the spiral ganglion. Studies have shown that tissues with a high energy demand are more susceptible to aging (Keithley, 2020). Furthermore, Ding et al. (2018) demonstrated that Na/K-ATPase activity decreased by approximately 80% in aged CBA/Caj mice. This reduction severely impacted the energy metabolism of the tissues and organs in the mice, which may contribute to the accelerated aging of high-energy-demanding tissues such as the cochlea (Figure 2).

### 4.3 Inflammation

**Figure 3:**
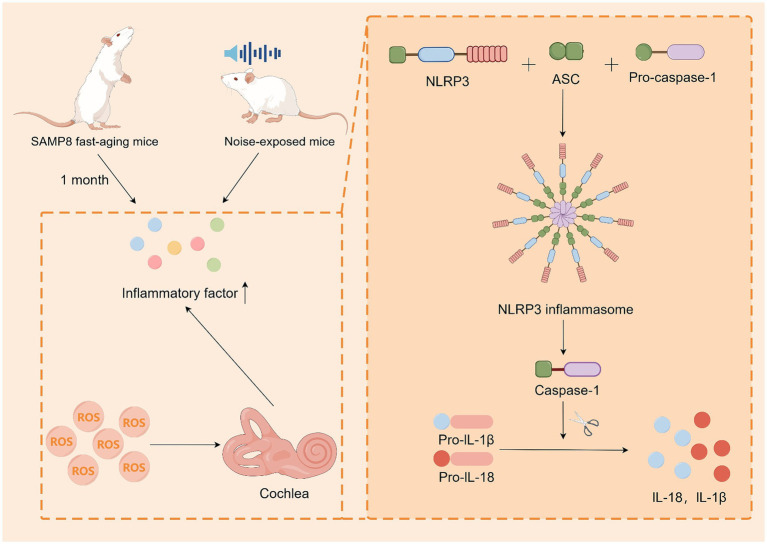
Inflammatory response may be one of the pathogenic mechanisms of ARHL. SAMP8 mice exhibited immune factor recruitment both at aging and in noise-exposed mice. Cellular experiments demonstrated that ROS induced cochlear cells to produce inflammatory vesicles, which regulated caspase-1-dependent activation of IL-1β and IL-18. By Figdraw.

Inflammation is a defensive mechanism of the body in the face of stimuli and is used to protect tissue cells and remove stimuli. Although acute inflammation can prevent damage and repair it, prolonged chronic inflammation can still cause damage to the body. With the development of modern medicine, chronic inflammation is closely associated with degenerative lesions in elderly individuals (Howcroft et al., 2013; Nash et al., 2014; Verschuur et al., 2012). Menardo et al. (2012) reported that SAMP8 fast-aging mice presented significant amounts of IL6-*β* and TNF-*α* in their cochlea after only 1 month, whereas R-line fast-aging resistant mice displayed this phenomenon only after 12–18 months. Tornabene et al. (2006) conducted a study on noise-exposed mice and reported that these mice presented increased expression of monocyte chemoattractant protein 5 (MCP-5), monocyte chemoattractant protein 1 (MCP-1), macrophage inflammatory protein-1β (MIP-1β), and intercellular adhesion molecule 1 (ICAM-1) compared with the unexposed group. These findings demonstrate that hearing damage induces immune cell recruitment. By examining the pathologic process of ARHL in senescent mice, Shi et al. (2017) demonstrated that hearing damage induces the recruitment of immune cells. They studied the cochlea and reported that the ROS-induced NLRP3 ROS sensor also binds to ASC to form inflammatory vesicles, which modulate the caspase-1-dependent activation of IL-1β and IL-18. This may lead to tissue damage and ARHL. These studies indicate that there is a close link between the inflammatory response and ARHL, as well as between the inflammatory response and oxidative reduction. This is likely one of the pathogenetic mechanisms of ARHL (Figure 3).

### 4.4 Oxidative stress

ROS and reactive nitrogen species (RNS) are produced mainly by mitochondria, the endoplasmic reticulum and peroxisomes under physiological and pathological conditions. ROS and RNS induce damage to DNA, lipids, and/or proteins *in vivo* with aging.

**Figure 4:**
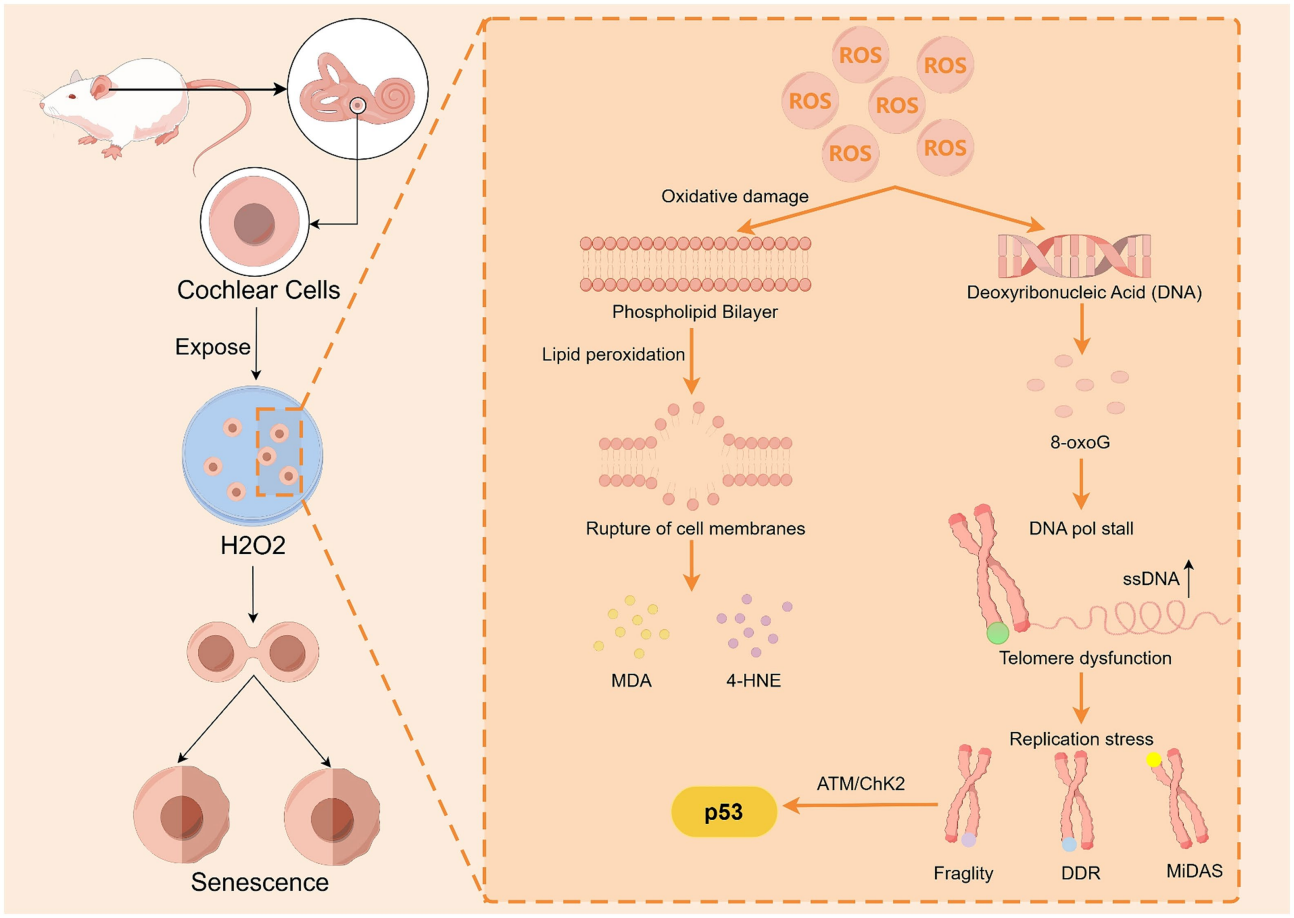
ROS accelerate cochlear cell senescence by damaging cell membrane lipids and DNA. Upon ROS damage, guanines in DNA are susceptible to 8-oxoG production, which leads to telomere fragility, localized DDR signaling, and MiDAS repair, thereby activating p53 to promote cellular senescence. ROS can cause lipid peroxidation of cell membranes, resulting in the production of MDA and 4-HNE, and MDA can cause secondary oxidative damage to proteins. By Figdraw.

The body has a range of antioxidant substances, such as superoxide dismutase (SOD), catalase (CAT), glutathione peroxidase (GPX), and glutathione reductase (GSR), that resist the damage caused by oxidative stress. However, as the body ages, this balance becomes dysfunctional, leading to mitochondrial damage, DNA damage, and cell apoptosis. In a study conducted by Menardo et al. (2012) on rapidly aging SAMP8 mice, the levels of the lipid peroxide malondialdehyde (MDA) in the cochlear tissues of SAMP8 mice were significantly greater than those in the cochlear tissues of SAMR1 mice at both 1 and 9 months of age, and MDA could cause secondary oxidative damage to proteins (Traverso et al., 2004). In addition, experiments were conducted to examine the levels of 8-oxoG in the cochleae of SAMP8 mice, a product of oxidative damage to guanine, which leads to telomere fragility, localized DNA damage response (DDR) signaling, and the repair of mitotic DNA repair synthesis (MiDAS) at telomeres, which in turn disrupts the normal cell division cycle without shortening the telomeres. 8-oxoG was shown to activate the ATM/ChK2 pathway within minutes, followed by further activation of p53 signaling, which prevents cell growth and accelerates premature senescence (Barnes et al., 2022). The results revealed that oxidative DNA damage in the cochleae of the SAMP8 mice was more severe than that in the control mice. Benkafadar et al. (2019) conducted experiments that demonstrated how hydrogen peroxide (H_2_O_2_) causes DNA damage in the cochlear hair cells of mice. After division, cochlear cells exposed to H_2_O_2_ exhibit significant senescence characteristics. However, the use of SOD and CAT could attenuate ARHL and hair cell damage in mice. Keithley et al. (2005) reported that SOD1 deficiency resulted in an age-related increase in cochlear hair cell loss, a decrease in vascular stripe thickness, and severe degeneration of spiral ganglion neurons in Cu/Zn superoxide dismutase (SOD1)-deficient mice. These findings suggest that redox reactions may be a significant mechanism for ARHL (Figure 4). In conclusion, oxidative stress may be closely related to the development of ARHL, and antioxidants can effectively protect cochlear tissue and attenuate ARHL.

## 5 Audiologic rehabilitation and treatment of ARHL

The *World Hearing Report 2021* identified three commonly used methods for AR in patients with hearing loss: artificial hearing technology, sign language and sensory substitution, and rehabilitation therapy (Chadha et al., 2021). Importantly, while AR can benefit patients diagnosed with hearing loss, not all methods are suitable for every patient due to the irreversibility of most hearing damage. Each rehabilitation strategy has its own strengths and weaknesses, and the patient should choose the one that best suits his or her situation, taking into account the AR resources available in the area. Detailed information can be found in Table 1. Patients with ARHL are typically middle-aged or older adults who may have poor metabolism, slow wound healing, and limited access to many medications and invasive therapies compared with younger adults. Therefore, interventions for ARHL should be based on conventional audiological rehabilitation and further standardized on the basis of conventional aural rehabilitation. Standardizing audiological rehabilitation is particularly important because of the poor screening rate, intervention rate, and cooperation rate of patients with ARHL in both clinical and social settings.

**Table 1:** Comparison of rehabilitation strategies.

	Invasive	Indications	Contraindications	Drawbacks
Artificial hearing technology				
Hearing aids	Non-invasive	Patients of all ages with partial hearing loss. Patients with predominantly high-frequency hearing loss and normal or near-normal low frequencies. Average hearing loss less than 90 dB.	Extremely severe sensorineural deafness patient. Total deafness, central and non-organic hearing loss. Foreign body or cerumen impaction in the ear. Vertigo symptoms. History of acute middle ear effusion within the last 3 months.	Limited lifespan, usually needing a new hearing aid within a few years. Prolonged wear may induce ear canal inflammation and cerumen blockage. Hearing aids amplify noise, further damaging hearing for ARHL patients.
Cochlear implant	Invasive	Congenital or acquired severe or profound sensorineural deafness. Even with hearing loss, the patient’s auditory nerve function remains intact and normal. Working better for early implantees (especially children). Improvements with cochlear implants are often more dramatic in patients with shorter periods of hearing loss.	A total loss of function of the auditory nerve. Active middle or inner ear infections. Patients with severe cognitive impairments or psychological problems may be unable to adapt to the auditory alterations associated with cochlear implants. Severe anatomical deformities of the ear.	Surgical risks such as infection, bleeding, anesthetic reactions. High cost of surgery and equipment maintenance. Patients need some time to adjust and learn after implantation, and some of them may face difficulties in adjusting and learning. The device may not be compatible with certain medical tests, such as MRI scans.
Bone conduction devices (non-implantable)	Non-invasive	Conductive hearing loss due to malformation of the outer or middle ear. An alternative for patients who cannot wear traditional hearing aids due to ear canal disease. Athletes and other individuals who regularly participate in water sports or other activities.	Skull defect or fracture. Active infection and ear inflammation. Severe middle ear pathology.	Compared to conventional hearing aids, BCDs are usually inferior in sound quality. Lack of clarity in high frequency sounds. Prolonged wear may cause discomfort and pressure sores. Open design leads to noise susceptibility.
Middle ear implants	Invasive	Patients with conductive hearing loss due to damage to middle ear structures. Have both conductive and sensorineural hearing loss. Chronic or severe ear drum perforation. Abnormal development of the external auditory canal or severe damage due to disease or accident.	Active otitis media or ear infections. Patients with severely impaired inner ear function. Complete loss of auditory nerve function.	Surgical risks such as infection, bleeding, anesthetic reactions. High cost of surgery and equipment maintenance. The device may not be compatible with certain medical tests, such as MRI scans.
Sign language and sensory substitution				
Sign language	Non-invasive	Total deafness or extreme hearing loss. Children with congenital hearing impairment.	It may not be the optimal form of communication in certain situations: Those who do not know sign language. hand injury or other motor impairment.	Grammar is unique and takes a long time to master for those who are weak learners. Lack of resources for sign language education. Low popularity of sign language in socialization.
Subtitle	Non-invasive	Patients with any level of hearing loss. Patients with hearing loss who communicate through spoken language.	Illiteracy Serious eye disease or after eye surgery. Permanent blindness or severe visual impairment due to optic nerve damage	In situations where dialog is frequent or spoken at a fast pace, it might be hard for people with hearing loss to read the subtitles in time. Presents a challenge in accurately translating language with specific cultural contexts or puns.
Rehabilitation				
Audiological rehabilitation	Non-invasive	Patients with any level of hearing loss of all ages.	Severe infection or inflammation in the ear. Total deafness. Recent Ear Surgery.	Difficulty in accessing quality audiological rehabilitation services in some remote areas. High variability in the level of audiological rehabilitation practitioners. Prolonged access to audiological rehabilitation services can put ongoing financial strain on patients.

### 5.1 Artificial hearing technology

#### 5.1.1 Hearing aids

HAs are commonly used as rehabilitation tools for patients of all ages with partial hearing loss. The basic principle of a HA is to amplify external sounds and compensate for the patient’s decreased hearing threshold, resulting in improved hearing levels and therapeutic effects. Currently, there are two main types of mainstreams HAs: analog and digital. A randomized controlled trial demonstrated that wearing HAs can significantly improve the quality of life related to hearing for elderly patients with hearing loss who engage in social activities. The researcher provided free HAs and guidance to the treatment group, whereas the control group only participated in the assessment. After a follow-up period of 20 months, the mood, mental state, cognition, and daily living ability of both groups were assessed and compared. The subjects with socialization showed significant improvement in hearing-related activities of daily living and anxiety-depression, whereas the effect was not significant in subjects with limited or no socialization (Ye et al., 2022). Anzivino et al. (2019) study reached a similar conclusion. The researchers followed 44 elderly deaf patients over 60 years of age who wore HAs for a period of 6–12 months. The study revealed improvements in short-term/long-term memory and cognitive and executive abilities. Additionally, there were significant differences between the subjects in terms of their physical and emotional impacts on their lives, general health vitality, and social activities.

In addition to benefiting users, it is important to consider the social and economic advantages of HAs. According to the WHO, the return on investment for unilateral HAs ranges from 1.62 to 1.84. This means that for every dollar invested, $60,138 in disability-adjusted life years (DALYs) can be saved for high-income populations and $3,564 for low-and middle-income populations (Chadha et al., 2021). At the same time, improving the hearing cognitive level of ARHL patients may also alleviate some of the social problems and family conflicts that currently exist, such as family conflicts caused by communication difficulties. However, as described at the outset, although research has demonstrated the benefits of being equipped with HAs and that HAs are the most well-known of the various rehabilitation strategies, the use of HAs is still low, and there is a lack of programs that can promote HAs on a large scale.

#### 5.1.2 Cochlear implants

Cochlear implants (CIs) are devices that convert acoustic signals into electrical signals and stimulates the auditory nerve to restore a patient’s hearing. It is considered one of the most successful neuroprostheses at all times. The main structure consists of an extraauricular machine that collects external acoustic signals and an intraauricular machine that stimulates the auditory nerve directly. The CIs are surgically implanted to directly stimulate the auditory nerve by bypassing the outer and middle ear, and are indicated for severe and profound bilateral sensorineural/conductive hearing loss (Michels et al., 2019). Traditionally, CIs have been used primarily to treat pediatric hearing loss. However, with recent advances in medicine, CIs have been increasingly used in older patients. Studies have shown that CIs are beneficial for speech perception, social functioning, and overall quality of life in elderly individuals (Hilly et al., 2016). Mosnier et al. (2015) and Mosnier et al. (2018) study demonstrated that cochlear implantation, coupled with auditory–communication rehabilitation, improved speech perception and cognition in elderly patients. Additionally, it had a positive effect on their social activities and quality of life. In patients with mild cognitive impairment, CIs can also help maintain or improve their cognitive ability (Andries et al., 2023). In addition to traditional CIs, a study of a newly proposed artificial CIs shows great potential. This study used bioinspired soft elastic metamaterials to reproduce the morphology and articulation of the human cochlea and successfully activated the auditory pathway in mice, opening up new avenues for patients with all types of hearing loss (Tang et al., 2023). Unlike HAs, although CIs have better therapeutic effects and wider ranges of indications, there are still many limitations on the use of CIs in elderly individuals due to its invasiveness, cost, and posttraining debugging requirements.

CIs also have significant economic benefits. Unilateral CIs implantation, for example, has an ROI of 1.46–4.09, with each dollar invested, saving $38,153 in disability adjusted life year (DALY) for high-income people and $6,907 for low-and middle-income people.

#### 5.1.3 Surgical implants

Bone conduction devices (BCDs) and middle ear implants (MEIs) are effective AR options for patients who cannot use HAs or CIs because of inflammation or structural deformities in the outer ear.

BCDs are classified as either implantable or nonimplantable. However, nonimplantable BCDs are often preferred because of their noninvasiveness and anatomical specificity requirements. The main principle of BCDs operation is to use solid-state vibration sound transmission by vibrating the skull to deliver sound directly to the inner ear, bypassing the middle ear. Reviews indicate that nonimplantable BCDs can be a safe and effective solution for patients with conductive or sensorineural hearing loss or unilateral deafness (Moffa et al., 2020). Furuta et al. (2022) developed a novel piezoelectric transducer with skin as the electrode for high-efficiency BCDs as a potential solution to side effects such as skin erosion and discomfort caused by conventional BCDs. Researchers have experimentally demonstrated its ability to achieve sound conduction with attenuated side effects, making it a viable alternative to current BCDs.

MEIs are classified as either semi-implantable or fully implantable HAs. Like CIs, MEIs consist of in-ear and out-of-ear devices. However, MEIs differ in that they transmit vibration energy directly to the auditory chain and cochlea through methods such as direct vibration and magnetic field vibration for therapeutic purposes. Some of the more commonly used MEIs systems include the Vibrant Soundbridge, Maxum, Carina, and Esteem systems (Bittencourt et al., 2014). A retrospective study revealed that bilateral complete implantation of MEIs is an effective therapeutic strategy for improving hearing and speech perception in noisy environments (Cuda et al., 2021).

#### 5.1.4 Hearing assistive devices

In addition to the aforementioned treatment strategies, hearing assistive devices can also improve the interaction ability of individuals with hearing loss. Mainstream hearing assistive devices currently include visual and tactile sensory cues, hearing enhancement in public places, and speech-to-text conversion devices. The primary objective of sensory alert devices is to capture the attention of hard-of-hearing patients through the use of sound, light, images, and vibration. Examples of such devices include vibrating bracelets, flashing alarms, and doorbell signals. Hearing enhancement devices in public places are based on traditional HAs. A signal transmission device is set up in public places, such as theaters, to transmit sound signals directly to the HAs of a patient with hearing loss. This effectively reduces environmental interference and improves the effectiveness of HAs. Speech-to-text conversion devices can receive surrounding acoustic signals, convert them into text, and present them to patients with hearing loss. This enhances their ability to interact with others.

### 5.2 Sign language and sensory substitution

Sign language is a form of communication commonly used by people who are deaf or hard of hearing. It involves using hand gestures to convey meaning and can be learned in as little as 3 months. Research has shown that using sign language can have a positive effect on the academic performance of deaf and hard of hearing children (Fitzpatrick et al., 2016; Marschark et al., 2006). There is currently no conclusive evidence to support the effectiveness of sign language in treating ARHL. However, owing to the advanced age and potential cognitive impairments of ARHL patients, the use of sign language as a potential treatment option should be further explored. Additionally, visual aids may also be a viable alternative to auditory assistance. The means to equip offline activities, such as meetings, performances, and video materials, with subtitles are currently more commonly used. This is an important means of information acquisition for patients with hearing loss who communicate through spoken language. Payne et al. (2022) study demonstrated that presenting textual subtitles simultaneously counteracted the negative effects of effortful listening on phonological memory. This resulted in improved memory for recognizing long sentences in older adults. This study highlights the importance of equipping older adults with subtitles to improve their information acquisition and environmental interaction, particularly those with hearing loss.

### 5.3 Rehabilitation

Audiological rehabilitation (AR) is a crucial treatment approach for all patients with hearing loss. It involves reducing deficits in function, activity, participation, and quality of life caused by hearing loss through sensory management, instruction, perceptual training, and counseling. Perceptual ability training can be used to maximize the use of a patient’s residual hearing or improve the fit between the patient and their hearing aid device. This can lead to better training in speech and communication abilities. Language training can be conducted in various ways, such as oral, sign language, lip reading, and multilingual teaching. Research has demonstrated that providing AR services to individuals who have already been fitted with HAs or providing such services prior to the fitting of HAs can effectively increase the use of HAs and improve the psychosocial well-being of users (Han et al., 2022; Bernstein et al., 2021). Moreover, in recent years, many minimally invasive or noninvasive techniques have been incorporated into AR, such as microbubble-assisted ultrasound, which combines ultrasound with microbubbles in the middle ear, and the new magnetic/acoustic dual-controlled microrobot, which can efficiently transport medications to the inner ear without damaging the ear tissues, making the treatment and rehabilitation of hearing impairment more effective (Yi et al., 2024; Micaletti et al., 2024). Although the importance of AR has been increasingly recognized, research on AR in recent years has focused mainly on tele-rehabilitation, some small surveys and cross-sectional studies, and high-quality prospective studies and randomized controlled trials (RCTs) with large sample sizes, as well as studies with high quality and impact factors, are lacking. Therefore, further research in this area is needed to provide more evidence (Scarinci et al., 2022; Koerber et al., 2022; Turunen-Taheri et al., 2019; Aazh and Moore, 2017; Saunders and Chisolm, 2015).

## 6 Conclusion

This paper presents a review of the clinical classification, etiology, and rehabilitation treatment options for ARHL, which is gaining attention as a common sensory disorder in elderly individuals because of the accelerating global aging process and increasing incidence of age-related diseases. ARHL can be classified into five types and composites based on differences in pathogenic mechanisms. Common causes include aging, genetics, noise exposure, ototoxic drugs, and metabolic diseases. AR options for ARHL patients include artificial hearing technology, sensory substitution, sign language, and rehabilitation training. Each method has its own advantages and disadvantages and should be used in conjunction with the patient’s condition to achieve the goal of social reintegration. Understanding the causes of ARHL, its clinical classification, and available rehabilitation strategies enables clinicians to develop specific treatment plans and guidance for ARHL patients, improving their quality of life.

The prevalence of ARHL, one of the most common sensory disorders in elderly individuals, is increasing as the population ages. Our understanding of the causative factors, mechanisms and interventions of ARHL is still limited compared with that of other types of hearing loss. In the future, research on the genetics of ARHL and new interventions will continue to be a hot topic in the field. Owing to the current lack of data on ARHL worldwide, large-scale epidemiological surveys should be conducted to screen for lifestyle habits, social factors, and diseases that are highly correlated with ARHL through statistical analyses. These findings provide a reference for the development of prevention and treatment strategies for ARHL that are tailored to the characteristics of elderly individuals.
